# Analysis of appendicitis management during COVID-19 pandemic: A study of Chinese adult cohorts

**DOI:** 10.3389/fsurg.2022.961258

**Published:** 2022-07-27

**Authors:** Wei-Di Wang, Jin-Quan Lin, Guang-Wei Zheng, Zhi-Peng Fang, Yi-Xing Yan

**Affiliations:** Trauma Center and Emergency Surgery Department, The First Affiliated Hospital of Fujian Medical University, Fuzhou, China

**Keywords:** COVID-19, acute appendicitis, complicated appendicitis, lockdown-period, health services

## Abstract

**Background:**

Healthcare seeking behavior has been widely impacted due to the restricted movements of individuals during the Coronavirus disease-19 (COVID-19) pandemic. This study aims to perform risk stratification in patients requiring timely intervention during the recovery periods.

**Methods:**

Operation notes of acute appendicitis (AA) patients within a hospital were analyzed during three six-month periods (23 January-23 July in 2019, 2020, and 2021, respectively). Patient data were collected retrospectively including demographics, pre-emergency status, perioperative information, postoperative outcomes, and follow-up results.

**Results:**

321 patients were included in this study, with 111, 86, and 124 patients in 2019, 2020, and 2021 groups, respectively. The median age of patients decreased by 4 years in 2020 as compared to that in 2019. The proportion of pre-hospitalization symptoms duration of more than 48 h in the 2020 group was higher (36.05% in 2020 vs. 22.52% in 2019). Length of hospital stay (LOS) in 2020 was shorter than it was during the same period in 2019 (4.77 vs. 5.64) and LOS in 2021 was shorter than in 2019 (4.13 vs. 5.64). Compared to the lockdown period, the proportion of patients with recurrent AA was higher in the post-lockdown period (15.1% vs. 27.4%). The median age was 34 years (vaccinated) vs. 37 years (unvaccinated). Logistic regression suggests that elevated C-reactive protein (CRP) (OR = 1.018, CI = 1.010–1.028), white cell count (WBC) (OR = 1.207, CI = 1.079–1.350), female (OR = 2.958, CI = 1.286–6.802), recurrent (OR = 3.865, CI = 1.149–12.997), and fecalith (OR = 2.308, CI = 1.007–5.289) were associated with complicated appendicitis (CA).

**Conclusion:**

The lockdown measures during the COVID-19 epidemic are shown to be correlated with a reduction in the proportion of AA patients who underwent surgery, particularly in older adults. Risk factors for CA include elevated CRP, WBC, female, recurrent, and fecalith.

## Introduction

In December 2019, the Coronavirus disease-19 (COVID-19) was first reported in China (1). On January 23, China imposed a series of nationwide lockdown measures to minimize inter-city travel and regional activities, including traffic restrictions, increased intersection checkpoints, compulsory self-quarantine at home, and shutdown of non-essential public facilities (2).

It has been shown that lockdown measures can effectively slow down the spread of the virus (3, 4). However, the “lockdown effect” has also impacted on treatment-seeking behavior of patients given the increased panic of the public to COVID-19 and the strict lockdown policy (5, 6). Indeed, the number of non-trauma surgical patients presenting to emergency departments decreased by more than 30% during the pandemic (7, 8). For patients with acute appendicitis (AA), the lockdown measures have caused a delay in seeking medical treatment. Such delay has been considered as a risk factor for complicated appendicitis (CA) (9, 10). The delayed medical support is also associated with an increased length of hospital stay (LOS) and increased total hospital cost (11). Without timely intervention, uncomplicated appendicitis (UA) may proceed to appendix gangrene, abscess, or perforation (12). Therefore, several studies have emphasized the impact of the “lockdown effect” on emergency services.

After several months of strict lockdown, the spread of the COVID-19 has been largely contained in China. In 2021, China entered a post-lockdown period featured by an increased vaccination rate (13). However, there is a lack of studies comparing the clinical characteristics and outcomes of patients who underwent appendectomy during these three periods, namely the pre-lockdown period (2019), the lockdown period (2020), and the post-lockdown period (2021). This study quantitatively analyzed the different clinical characteristics and outcomes of patients undergoing appendectomy during the three periods, respectively. In addition, the impact of COVID-19 vaccination on patients who underwent appendectomy and the potential risk factors for CA was also explored. The outcomes of this study are expected to provide an effective management strategy for patients with AA during the sudden pandemic of an infectious disease.

## Population and methods

No informed consent was obtained given that this was a retrospective study. The study was reviewed and approved by the Institutional Review Board at The First Affiliated Hospital of Fujian Medical University (Approval No IEC-FOM-013-2.0). The study was conducted in a teaching tertiary hospital, which is at the forefront of clinical practice, scientific research, and medical education in China.

## Data collection

Data were collected from patients with postoperative pathology showing AA and who underwent appendectomy (open or laparoscopic) during a six-month period from 23 January to 23 July 2020. Collected data were compared with AA patients in similar conditions during the same period in 2019 and 2021. All patients were informed about the surgical procedures, medications, anticipated outcomes, potential complications, and alternative treatment options (antibiotics/endoscopic appendix intubation and irrigation); all provided informed consent before surgery. None of the patients in this study were infected with COVID-19 virus which was confirmed by nucleic acid detection. All patients had a preoperative computed tomography (CT) scan. The 30-day follow-up was performed by an independent reviewer by telephone when relevant information was also obtained.

Exclusion criteria included: (1) patients with other acute abdomen diseases, such as ureteral calculus, ileocecal diverticulitis, etc. (*n* = 4); (2) patients were suspected to have appendiceal tumors (*n* = 2); (3) patients who underwent other surgery during the same period (*n* = 5); (4) age <18 (*n* = 8).

The collected data includes demographics, duration of pre-hospitalization symptoms, American Society of Anesthesiologists risk score(ASA score) (14), preparative times of pre-operation, operation time, surgical findings, surgical approach, LOS, postoperative complications, pathology reports, 30-day readmission rate, and vaccination status(receive at least one dose of vaccination). The appendicitis inflammatory response (AIR) scores were calculated based on the collected data (15).

In general, AA is divided into two categories: UA (suppurative/phlegmonous) and CA (gangrenous/perforated/abscess) based on the intraoperative observation and pathological types (16). None of the patients were found to have an appendiceal abscess in this study given that patients with an appendiceal abscess in our hospital would mainly undergo ultrasound-guided percutaneous catheter drainage and would receive intravenous antibiotics.

Recurrent was defined as a relapse in which the patient had received non-operative treatment in the past and was now recurrent. In terms of the study periods, 2019 (23 January to 23 July), 2020, and 2021 were defined as the pre-lockdown period, the lockdown period, and the post-lockdown period, respectively.

## Statistical analysis

To compare the clinical characteristics and outcomes of AA patients in the 2019, 2020, and 2021 groups, categorical variables were analyzed using the Chi-square test or Fisher's exact test, and continuous variables were analyzed using the t test. The age data were summarized by median and inter-quartile range (IQR) and analyzed with the Mann-Whitney U test as the data did not match normal distribution. The effect of each variable on the rates of CA was assessed using a binary logistic regression test. Statistical analyses were performed using Statistical Package for Social Sciences™ (SPSS™) version 23. A p-value less than 0.05 was considered as statistically significant.

## Results

321 patients were included in this study, with 111, 86, and 124 patients in 2019, 2020, and 2021 groups, respectively.

### 2019 vs. 2020

#### Demographic and clinical characteristics

The number of patients who underwent appendectomy decreased by 22.5% in 2020 compared with the number in 2019 (86 in 2020vs. 111 in 2019). There was no significant difference in sex, white cell count (WBC), C-reactive protein (CRP) level, neutrophile granulocyte rate (*N*%), body temperature, pulse, body-mass index (BMI), obesity rate, ASA score, and preoperative preparation time. It is worth mentioning that the duration of pre-hospitalization symptoms of 2020 group was slightly higher than that of 2019 group but not statistically significant. This was further supported by the significantly higher proportion of pre-hospitalization symptoms duration of more than 48 h in the 2020 group (36.05% in 2020 vs. 22.52% in 2019, *p* = 0.037).

**Figure 1:**
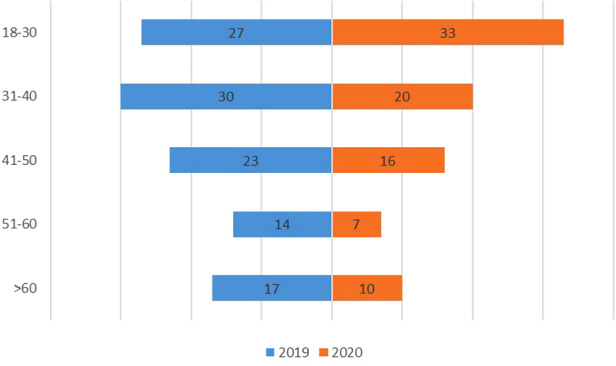
Comparisons of age distribution of AA patients in 2019 and 2020. The box plot indicates the number of AA patients in 2019 (blue) and 2020 (orange).

The median age of the patients decreased by 4 years in 2020 as compared to that in 2019 (36 in 2020 vs. 40 in 2019, *p* = 0.042) with significant differences in the age distribution (see Figure 1). Of note, the AIR scores significantly increased from 4.63 in 2019 to 5.45 in 2020 (*p* = 0.013). The proportion of mild and high probability was slightly higher in the 2020 cohort than in 2019, which nearly reached statistically significant (*p* = 0.050). The clinical characteristics of patients are summarized in Table 1.

**Table 1:** Comparisons of baseline demographic and clinical characteristics of patients.

Characteristic	January–July 2019 Group (*n* = 111)	January–July 2020 Group (*n* = 86)	*p*	January–July 2020 Group (*n* = 86)	January–July 2021 Group (*n* = 124)	*p*	January–July 2019 Group (*n* = 111)	January–July 2021 Group (*n* = 124)	*p*
Age (years), median IQR (1/4)	40.0 (31.0, 52.0)	36 (25.0, 47.0)	0.042	36 (25.0, 47.0)	35 (26.0, 52.0)	0.638	40.0 (31.0, 52.0)	35 (26.0, 52.0)	0.140
Female, *n* (%)	50 (45.05)	40 (46.51)	0.838	40 (46.51)	61 (49.20)	0.702	50 (45.05)	61 (49.20)	0.525
Pre-hospitalization symptoms duration (hours), mean ± SD	30.73 (20.47)	35.37 (18.70)	0.103	35.37 (18.70)	32.51 (20.43)	0.302	30.73 (20.47)	32.51 (20.43)	0.506
Pre-hospitalization symptoms duration >48 h, *n* (%)	25 (22.52)	31 (36.05)	0.037	31 (36.05)	37 (29.84)	0.344	25 (22.52)	37 (29.84)	0.204
Preoperative preparation time (h), mean ± SD	7.44 (5.66)	6.54 (5.37)	0.258	6.54 (5.37)	6.47 (4.69)	0.970	7.44 (5.66)	6.47 (4.69)	0.150
ASA I, *n* (%)	85 (76.6)	65 (75.6)	0.915	65 (75.6)	94 (75.8)	0.974	85 (76.6)	94 (75.8)	0.881
ASA II, *n* (%)	22 (19.8)	19 (22.1)		19 (22.1)	25 (20.2)		22 (19.8)	25 (20.2)	
ASA III, *n* (%)	4 (3.6)	2 (2.3)		2 (2.3)	5 (4.0)		4 (3.6)	5 (4.0)	
WBC (×10^9^/L), mean ± SD	10.229 (4.28)	11.399 (5.79)	0.118	11.399 (5.79)	10.80 (4.69)	0.410	10.229 (4.28)	10.80 (4.69)	0.332
CRP (mg/L), mean ± SD	55.21 (57.61)	61.66 (62.15)	0.453	61.66 (62.15)	51.38 (50.62)	0.206	55.21 (57.61)	51.38 (50.62)	0.588
*N* (%), mean ± SD	75.38 (14.06)	77.552 (14.67)	0.294	77.552 (14.67)	78.01 (14.97)	0.827	75.38 (14.06)	78.01 (14.97)	0.169
Body temperature (°C), mean ± SD	36.71 (0.60)	36.73 (0.54)	0.846	36.73 (0.54)	36.67 (0.40)	0.326	36.71 (0.60)	36.67 (0.40)	0.477
Pulse, mean ± SD	82.96 (12.96)	84.07 (16.77)	0.602	84.07 (16.77)	82.31 (12.52)	0.386	82.96 (12.96)	82.31 (12.52)	0.697
BMI (kg/m^2^), mean ± SD	22.916 (3.45)	22.431 (3.35)	0.323	22.431 (3.35)	22.299 (3.24)	0.775	22.916 (3.45)	22.299 (3.24)	0.159
Obesity (body mass index >25 kg/m^2^), *n* (%)	29 (26.1)	17 (19.8)	0.295	17 (19.8)	26 (21.0)	0.832	29 (26.1)	26 (21.0)	0.351
Recurrent, *n* (%)	20 (18.0)	13 (15.1)	0.589	13 (15.1)	34 (27.4)	0.035	20 (18.0)	34 (27.4)	0.087
AIR Score, mean ± SD	4.63 (2.17)	5.45 (2.44)	0.013	5.45 (2.44)	5.28 (2.54)	0.626	4.63 (2.17)	5.28 (2.54)	0.035
Low probability, *n* (%)	54 (48.6)	32 (37.2)	0.050	32 (37.2)	49 (39.5)	0.867	54 (48.6)	49 (39.5)	0.055
Mild probability, *n* (%)	52 (46.8)	44 (51.2)		44 (51.2)	59 (47.6)		52 (46.8)	59 (47.6)	
High probability, *n* (%)	5 (4.5)	10 (11.6)		10 (11.6)	16 (12.9)		5 (4.5)	16 (12.9)	

IQR, interquartile range; AIR, appendicitis inflammatory response; WBC, white cell count; CRP, C-reactive protein; N %, neutrophile granulocyte rate; BMI, body-mass index; ASA, American society of anesthesiologists.

#### Finding in surgery and clinical outcome

The ratio of patients who underwent a laparoscopic appendectomy in 2019 was similar to that in 2020. In the latter, only one patient changed from laparoscopic appendectomy to open appendectomy due to peritoneal adhesions. According to the collected follow-up data, none of the patients underwent reoperation within one month. LOS in 2020 (4.77) was shorter than that in 2019 (5.64, *p* = 0.017). Analysis of pathology revealed that the rate of patients with an appendiceal fecalith was similar between the two groups as well as the mean maximum diameter and the length of the appendix. The average surgical duration during lockdown period was slightly higher than that during pre-lockdown period (72.89 vs. 69.25, *p* = 0.465). In addition, no statistically significant difference was found in the rate of CA cases, postoperative complications, and 30-day readmission rate between 2019 and 2020 groups (Table 2).

**Table 2:** Comparisons of clinical outcomes.

Outcomes	January–June 2019 Group (*n*= 111)	January–June 2020 Group (*n* = 86)	*p*	January–June 2020 Group (*n* = 86)	January–June 2021 Group (*n* = 124)	*p*	January–June 2019 Group (*n* = 111)	January–June 2021 Group (*n* = 124)	*p*
Laparoscopic appendectomy, *n* (%)	107 (96.4%)	79 (91.9%)	0.288	79 (91.9%)	119 (96.0)	0.338	107 (96.4%)	119 (96.0)	0.864
Laparoscopy converted to open appendectomy, *n* (%)	0	1 (1.2%)	0.437	1 (1.2%)	0	0.410	0	0	-
Complicated appendicitis, *n* (%)	17 (15.3%)	16 (18.6%)	0.540	16 (18.6%)	22 (17.7)	0.873	16 (14.4%)	22 (17.7)	0.618
Operation time (minutes), mean ± SD	69.25 (31.15)	72.89 (38.70)	0.465	72.89 (38.70)	76.54 (44.70)	0.432	69.25 (31.15)	76.54 (44.70)	0.153
Max diameter (mm), mean ± SD	10.53 (6.81)	10.44 (3.78)	0.913	10.44 (3.78)	10.52 (3.41)	0.882	10.53 (6.81)	10.52 (3.41)	0.982
Length of appendix (mm), mean ± SD	56.47 (14.31)	58.17 (16.39)	0.440	58.17 (16.39)	56.81 (16.02)	0.547	56.47 (14.31)	56.81 (16.02)	0.869
Fecalith, *n* (%)	39 (35.14%)	28 (32.56%)	0.705	28 (32.56%)	37 (29.8)	0.675	39 (35.14%)	37 (29.8)	0.386
Length of hospital stay (days), mean ± SD	5.64 (2.25)	4.77 (2.83)	0.017	4.77 (2.83)	4.13 (3.09)	0.129	5.64 (2.25)	4.13 (3.09)	<0.001
Postoperative complications, *n* (%)	3 (2.70)	3 (3.50)	0.532	3 (3.49)	2 (1.6)	0.677	3 (2.70)	2 (1.6)	0.900
30-day readmission, *n* (%)	0	1 (1.2)	0.437	1 (1.16)	0	0.410	0	0	–

### 2020 vs. 2021

There was no statistically significant difference in demographic and clinical characteristics of patients between the two groups. Postoperative complications and clinical outcomes did not differ significantly between the two groups (Table 2).

### 2019 vs. 2021

The AIR score was significantly higher in 2021 as compared to the 2019 group (5.28 vs. 4.63, *p* = 0.035). There was no significant difference in other demographic and clinical characteristics (Table 1). It is worth noting that the average LOS was significantly shorter in 2021 than it was in 2019 (4.13 vs. 5.64, *p* < 0.001) (Table 2).

#### Association between vaccination and clinical characteristics/outcomes

To further analyze the impact of vaccinations, patients during the post-lockdown period were classified into a vaccinated group and an unvaccinated group. In the vaccinated group, only three pathology reports showed CA (7.89%), while 19 cases of CA (22.09%) were reported in unvaccinated group.

**Figure 2:**
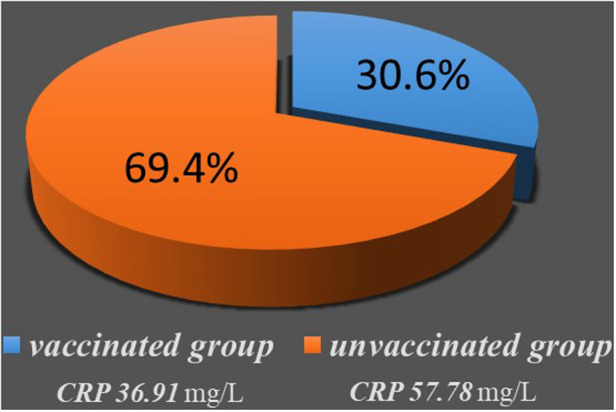
Comparisons of CRP value of AA patients between vaccinated group and unvaccinated group. Pie chart represent CRP value and the proportions at two groups, and chart area is proportional to the number of patients.

Pre-hospitalization symptoms duration was 34.28 h in the unvaccinated group whereas it was 27.24 h in the vaccinated group (*p* = 0.080). Patients in vaccinated group were younger than the patients in the vaccinated group (median age 34, IQR (25–47) vs. 37 IQR (28.5–52), *p* < 0.001). The mean CRP was significantly higher in the unvaccinated group than it was in the vaccinated group (57.78 vs. 36.91 *p* = 0.011) (Figure 2). No statistically significant difference was found in other clinical features/outcomes between the two groups (Table 3).

**Table 3:** Comparison of the vaccinated group and the non-vaccinated group.

Outcomes	Vaccinated Group (*n* = 38)	Unvaccinated Group (*n* = 86)	*p*
Age (years), median IQR(1/4)	34.0 (25, 47)	37 (28.5, 52)	<0.001
Female, *n* (%)	15 (39.5)	46 (53.5)	0.150
Pre-hospitalization symptoms duration (hours), mean ± SD	28.50 (13.37)	34.28 (22.70)	0.080
Pre-hospitalization symptoms duration >48 h, n (%)	8 (22.1)	29 (33.7)	0.155
WBC (×10^9^/L), mean ± SD	10.89 (4.20)	10.76 (4.91)	0.885
CRP (mg/L), mean ± SD	36.91 (33.56)	57.78 (55.52)	0.011
*N* (%), mean ± SD	78.18 (14.38)	77.93 (15.30)	0.934
Body temperature (°C), mean ± SD	36.67 (0.37)	36.67 (0.42)	0.925
Pulse, mean ± SD	85.53 (12.68)	80.90 (12.25)	0.057
BMI (kg/m^2^), mean ± SD	22.63 (3.65)	22.15 (3.05)	0.454
AIR score, mean ± SD	5.18 (2.17)	5.33 (2.70)	0.757
Recurrent, *n* (%)	6 (15.8)	28 (32.6)	0.054
Complicated appendicitis, *n* (%)	3 (7.9%)	19 (22.1%)	0.056
Postoperative complications, *n* (%)	1 (2.6)	1 (1.2)	0.521

IQR, interquartile range; AIR, appendicitis inflammatory response; WBC, white cell count; CRP, C-reactive protein; N%, neutrophile granulocyte rate; BMI, body-mass index; AIR score, appendicitis inflammatory response score.

#### Risk factors for CA

**Figure 3:**
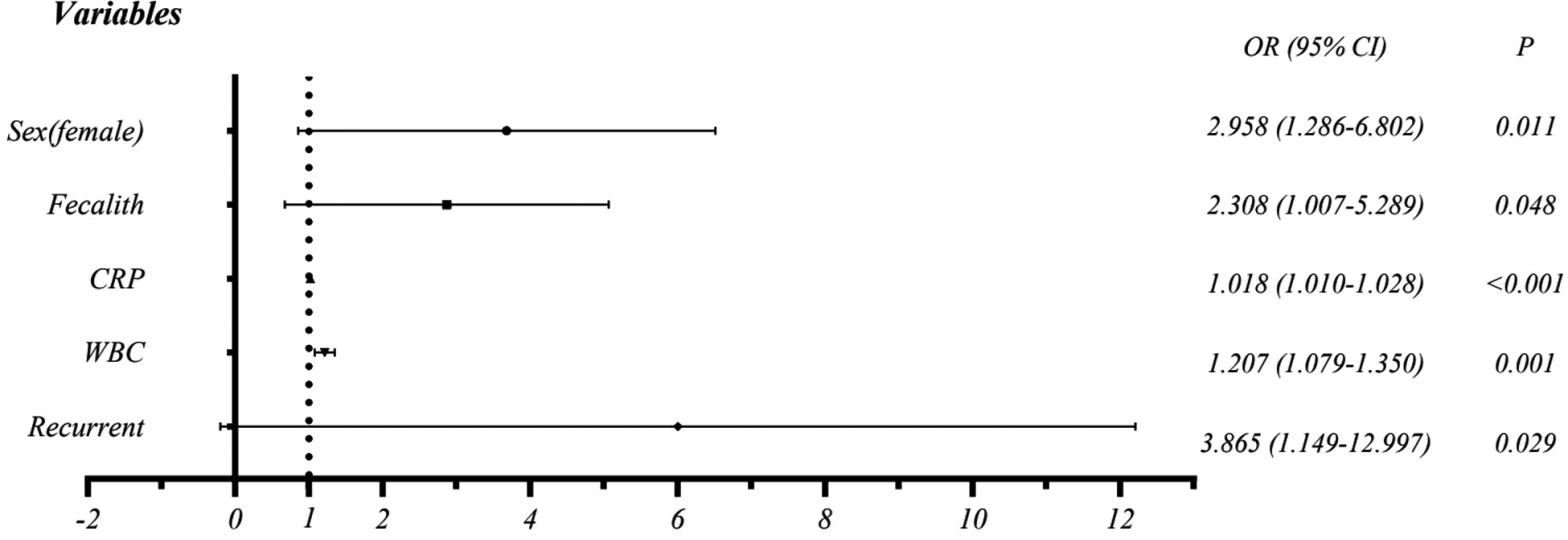
Forest plot of the risk factors for complicated appendicitis.

Binary logistic regression analysis was performed to identify variables that are significantly associated with CA. As a results, elevated CRP (OR = 1.018, CI = 1.010–1.028), WBC (OR = 1.207, CI = 1.079–1.350), female (OR = 2.958, CI = 1.286–6.802), recurrent (OR = 3.865, CI = 1.149–12.997), and fecalith (OR = 2.308, 1.007–5.289) were identified to be associated with CA (Table 4 and Figure 3).

**Table 4:** Multivariate analysis of demographic and clinical characteristics associated with CA.

Variable	*B*	*p* value	OR	95% CI
CRP	0.022	<0.001	1.018	1.010–1.028
WBC	0.186	0.001	1.207	1.079–1.350
Female	1.146	0.011	2.958	1.286–6.802
Fecalith	0.871	0.048	2.308	1.007–5.289
Recurrent	1.407	0.029	3.865	1.149–12.997

WBC, white cell count; CRP, C-reactive protein; OR, Exp (B).

## Discussion

Globally, governments have generally adopted social restrictions such as lockdown and “stay at home” orders in response to COVID-19 outbreak. The latter is likely to adversely affect the surgical treatment of patients.

The lack of postoperative pathology may lead to false positives in outpatients. Given the large total number of outpatients in the investigated institution, the number of patients with false positives may cause a bias. Therefore, this study focuses on the impact of the lockdown on patients who underwent appendectomy with particular importance attached to the outcomes and treatment-seeking behavior in AA surgical patients.

Our analysis demonstrated a significant decrease in the number of patients who received appendectomy during the COVID-19 outbreak compared to the pre-lockdown period. This is possibly due to the trepidation of patients towards COVID-19 infections in hospitals. Thus, patients with mild symptoms were inclined to choose conservative treatment (oral or intravenous antibiotics) in the community.

Furthermore, this study also shows a significant reduction in older adults who underwent surgery, suggesting that the pandemic may diminish the willingness of AA patients to undergo surgery as well as emergency hospitalization. Given that symptoms of elderly AA patients are atypical and susceptible to appendiceal gangrene and perforation, there is no evidence showing that the actual incidence of appendicitis decreased during the lockdown period. This suggests that lockdown measures prompt the elderly AA patients to delay their treatment or to choose conservative treatment. Consequently, these patients may miss the optimal surgical timing. Accordingly, the medical workers are expected to adjust management strategies in elderly patients with AA during similar lockdown periods.

As the pandemic entered a well-controlled stage in China (post-lockdown period in 2021), the number of patients who underwent appendectomy from 23 January to 23 July 2021 has significantly increased compared with the number in 2019 and 2020. This may reflect the effective control of the COVID-19 epidemic. A prospective study reported the overall recurrence rate of non-operatively treated AA with 2 years of follow-up was 13.8%. Such a recurrence rate is similar to that of our study during the period of 2019, and 2020, and was significantly lower than our proportion of recurrent appendicitis in 2021 (27.45%).

This result may be due to the population included in our study being patients who underwent surgery, and many patients with effective nonoperative treatment and no recurrence were excluded. Second, the remission of the epidemic has improved the patients' willingness to be hospitalized.

The COVID-19 pandemic outbreak has been shown to cause reluctance of patients to seek health care which results in an increased proportion of patients that received delayed medical services compared with the pre-lockdown period (17). The World Society of Emergency Surgery Jerusalem guideline suggests that pre-hospital delay is an independent predictor of CA, which may result in unnecessary morbidity and increased mortality (18). However, there is a controversial proportion of CA during the lockdown period.

The severity of many surgical diseases is considered to be linked to the length of pre-treatment duration, i.e., a delay in treatment may lead to severe complications (12). Indeed, previous studies have confirmed the increased disease severity in appendicitis patients during the lockdown period (19–21). Alberto Sartori et al. observed an increase in the number of post-operative complications in 2020 compared to 2019 (22). A META analysis that included 54 studies found the pandemic has altered the rate of admissions for AA and appendectomy, with parallel increased incidence of complicated cases in all age groups (23). The differences between these studies and our results may stem from the stringency of lockdown measures and the cost of surgery in different countries. The severity of the COVID-19 epidemic influenced patients' willingness to seek health care, leading to different delays in different countries. It is also worth noting that CA is not the only endpoint of untreated UA (24, 25). In addition, complicated and non-complicated appendicitis can be considered as two different disease entities. As shown in another work, a 24-h delay did not lead to a higher incidence of postoperative complications (26). Furthermore, Pedziwiatr et al. also pointed out other risk factors for complicated appendicitis such as gender, obesity, age >50 years, symptoms duration > 48 h, high Alvarado score, CRP > 100 mg/L (27). Similarly, Yaron et al. found that age, BMI, and duration of symptoms before emergency were important risk factors for complicated AA (28).

Although no difference was found in pre-hospitalization duration between 2019 and 2020 groups, the results show a significantly increased rate of prehospital delay (over 48 h) in the 2020 group while the rate of CA was similar in the two groups. It has been suggested that UA may not require drug or surgical intervention to recover. In addition, disruptions in use of surgical intervention treatment may not necessarily lead to an increase in CA (17, 29). According to the result in this work, CA was associated with female, elevated CRP, WBC, and fecalith, but not with preoperative preparation time or pre-hospitalization symptoms duration. Therefore, it is expected that patients with CA could have different clinical characteristics compared with UA patients. Furthermore, AA may not necessarily develop to CA, although patients with specific clinical characteristics (female, recurrent, fecalith, elevated CRP or WBC) tend to develop CA.

The clinical characteristics of the patients varied between CA patients and UA patients. However, the prognosis of patients is not distinct in postoperative complications and 30-day re-admission rate. This is also supported by the fact that the impact of lockdown on AA patients primarily lies in treatment-seeking behavior and psychology but not on prognosis. Considering that AA is a local inflammatory disease, fatal sequelae are unlikely to occur once excised. As a result, it is challenging to clarify the effect of lockdown on the rate of CA and the prognosis of AA with current data. Further prospective studies with a larger sample size to is required to clarify their correlation.

The LOS during the lockdown period significantly decreased, which reflects the concerns that hospitalization may expose the patients to COVID-19. The concern of the public could also account for the decreased number of people receiving surgery. Meanwhile, this study suggests that the vaccinated group had a younger median age and shorter delay, which may lead to differences in CRP and intraoperative findings between vaccinated and unvaccinated patients.

The limitations of this study are its retrospective nature and a relatively medium-sized single-center cohort. These may result in a specific bias in the data selection. In addition, the findings present in this work are valuable for the management of surgical AA patients in developing countries, but they may not be applicable in developed countries. Given that appendicitis is simply classified into CA or UA, subtle changes within other subcategories may not be distinguished with current methods. Moreover, long-term follow-up data relating to the prognosis of patients who received surgery is not available as the complications of appendicitis surgery are mostly present in the early stage (16).

## Conclusion

The lockdown measures during the COVID-19 pandemic have resulted in a reduced number of AA patients receiving surgery, particularly older adults. Binary regression analysis suggests that the risk factors for complicated AA include CRP, WBC, female, recurrent, and fecalith. Not all AA would develop to CA, but AA patients with specific clinical characteristics (elevated WBC or CRP, recurrent, female, and fecalith) would develop to CA. The outcomes of this work provide a practical reference for decision-makers in health services to develop targeted medical management during sudden pandemic of infectious diseases.

## Data Availability

The raw data supporting the conclusions of this article will be made available by the authors, without undue reservation.

## References

[1] Wang C Horby P Hayden F Gao G. A novel coronavirus outbreak of global health concern. Lancet. (2020) 395(10223):470–73. 10.1016/s0140-6736(20)30185-9 31986257 PMC7135038

[2] Tian H Liu Y Li Y Wu CH Chen B Kraemer MUG An investigation of transmission control measures during the first 50 days of the COVID-19 epidemic in China. Science. (2020) 368(6491):638–42. 10.1126/science.abb6105 PMC7164389 32234804

[3] Lau H Khosrawipour V Kocbach P Mikolajczyk A Schubert J Bania J The positive impact of lockdown in Wuhan on containing the COVID-19 outbreak in China. J Travel Med. (2020) 27(3):taaa037. 10.1093/jtm/taaa037 PMC7184469 32181488

[4] Wilder-Smith A Freedman D. Isolation, quarantine, social distancing and community containment: pivotal role for old-style public health measures in the novel coronavirus (2019-nCoV) outbreak. J Travel Med. (2020) 27(2):taaa020. 10.1093/jtm/taaa020 PMC7107565 32052841

[5] Pellegrini M Roda M Lupardi E Di Geronimo N Giannaccare G Schiavi C. The impact of COVID-19 pandemic on ophthalmological emergency department visits. Acta Ophthalmol. (2020) 98(8):e1058–e1059. 10.1111/aos.14489. 32483929 PMC7300851

[6] Lazzerini M Barbi E Apicella A Marchetti F Cardinale F Trobia G. Delayed access or provision of care in Italy resulting from fear of COVID-19. Lancet. (2020) 4(5):e10–e11. 10.1016/s2352-4642(20)30108-5 PMC7146704 32278365

[7] English W Habib Bedwani N Smith C Shatkar V. Investigation and management of suspected appendicitis during the COVID-19 pandemic. Br J Surg. (2020) 107(9):e337–e338. 10.1002/bjs.11787 32658307 PMC7404757

[8] Anteby R Zager Y Barash Y Nadler R Horesh N. The impact of the coronavirus sisease 2019 outbreak on the attendance of patients with surgical complaints at a tertiary hospital emergency department. J Laparoendosc Adv Surg Tech. (2020) 30(9):1001–7. 10.1089/lap.2020.0465 32589496

[9] Naderan M Babaki A Shoar S Mahmoodzadeh H Nasiri S Khorgami Z. Risk factors for the development of complicated appendicitis in adults. Ulus Cerrahi Derg. (2016) 32(1):37–42. 10.5152/ucd.2015.3031 26985166 PMC4771424

[10] Ditillo M Dziura J Rabinovici R. Is it safe to delay appendectomy in adults with acute appendicitis? Ann Surg. (2006) 244(5):656–60. 10.1097/01.sla.0000231726.53487.dd 17060754 PMC1856602

[11] Aiken T Barrett J Stahl C Schwartz P Udani S Acher A Operative delay in adults with appendicitis: time is money. J Surg Res. (2020) 253:232–37. 10.1016/j.jss.2020.03.038 32387570

[12] Lee-Archer P Blackall S Campbell H Boyd D Patel B McBride C. Increased incidence of complicated appendicitis during the COVID-19 pandemic. J Paediatr Child Health. (2020) 56(8):1313–14. 10.1111/jpc.15058 32830891 PMC7461078

[13] Wang J Jing R Lai X Zhang H Lyu Y Knoll MD Acceptance of COVID-19 vaccination during the COVID-19 pandemic in China. Vaccines (Basel). (2020) 8(3):482. 10.3390/vaccines8030482 PMC7565574 32867224

[14] Fransvea P Fico V Cozza V Costa G Lepre L Mercantini P Clinical-pathological features and treatment of acute appendicitis in the very elderly: an interim analysis of the FRAILESEL Italian multicentre prospective study. Eur J Trauma Emerg Surg. (2022) 48(2):1177–88. 10.1007/s00068-021-01645-9 33738537

[15] Kollár D McCartan DP Bourke M Cross KS Dowdall J. Predicting acute appendicitis? A comparison of the alvarado score, the appendicitis inflammatory response score and clinical assessment. World J Surg. (2015) 39(1):104–9. 10.1007/s00268-014-2794-6 25245432

[16] Bhangu A Søreide K Di Saverio S Assarsson J Drake F. Acute appendicitis: modern understanding of pathogenesis, diagnosis, and management. Lancet. (2015) 386(10000):1278–87. 10.1016/s0140-6736(15)00275-5 26460662

[17] Tankel J Keinan A Blich O Koussa M Helou B Shay S The decreasing incidence of acute appendicitis during COVID-19: a retrospective multi-centre study. World J Surg. (2020) 44(8):2458–63. 10.1007/s00268-020-05599-8 32458019 PMC7250252

[18] Di Saverio S Podda M De Simone B Ceresoli M Augustin G Gori A Diagnosis and treatment of acute appendicitis: 2020 update of the WSES Jerusalem guidelines. World J Emerg Surg. (2020) 15(1):27. 10.1186/s13017-020-00306-3 32295644 PMC7386163

[19] Romero J Valencia S Guerrero A. Acute appendicitis during coronavirus disease 2019 (COVID-19): changes in clinical presentation and CT findings. J Am Coll Radiol. (2020) 17(8):1011–13. 10.1016/j.jacr.2020.06.002 32610104 PMC7321660

[20] Dick L Green J Brown J Kennedy E Cassidy R Othman S Changes in emergency general surgery during COVID-19 in Scotland: a prospective cohort study. World J Surg. (2020) 44(11):3590–94. 10.1007/s00268-020-05760-3 32860140 PMC7454130

[21] Orthopoulos G Santone E Izzo F Tirabassi M Pérez-Caraballo A Corriveau N Increasing incidence of complicated appendicitis during COVID-19 pandemic. Am J Surg. (2021) 221(5):1056–60. 10.1016/j.amjsurg.2020.09.026 33012500 PMC7521886

[22] Sartori A Podda M Botteri E Passera R Agresta F Arezzo A. Appendectomy during the COVID-19 pandemic in Italy: a multicenter ambispective cohort study by the Italian Society of Endoscopic Surgery and new technologies (the CRAC study). Updates Surg. (2021) 73(6):2205–13. 10.1007/s13304-021-01126-z 34219197 PMC8255092

[23] Grossi U Gallo G Ortenzi M Piccino M Salimian N Guerrieri M Changes in hospital admissions and complications of acute appendicitis during the COVID-19 pandemic: a systematic review and meta-analysis. Health Sci Rev. (2022) 3:100021. 10.1016/j.hsr.2022.100021 PMC8906891 35287332

[24] Andersson R. The natural history and traditional management of appendicitis revisited: spontaneous resolution and predominance of prehospital perforations imply that a correct diagnosis is more important than an early diagnosis. World J Surg. (2007) 31(1):86–92. 10.1007/s00268-006-0056-y 17180556

[25] Livingston E Woodward W Sarosi G Haley R. Disconnect between incidence of nonperforated and perforated appendicitis: implications for pathophysiology and management. Ann Surg. (2007) 245(6):886–92. 10.1097/01.sla.0000256391.05233.aa 17522514 PMC1876946

[26] van Dijk S van Dijk A Dijkgraaf M Boermeester M. Meta-analysis of in-hospital delay before surgery as a risk factor for complications in patients with acute appendicitis. Br J Surg. (2018) 105(8):933–45. 10.1002/bjs.10873 29902346 PMC6033184

[27] Pedziwiatr M Lasek A Wysocki M Mavrikis J Mysliwiec P Bobowicz M Laparoscopic appendectomy: complicated appendicitis: risk factors and outcomes of laparoscopic appendectomy – polish laparoscopic appendectomy results from a multicenter, large-cohort study. Ulusal travma ve acil cerrahi dergisi. (2019) 25(2):129–36. 10.5505/tjtes.2018.80103 30892680

[28] Rudnicki Y Soback H Mekiten O Lifshiz G Avital S. The impact of COVID-19 pandemic lockdown on the incidence and outcome of complicated appendicitis. Surg Endosc. (2022) 36(5):3460–6. 10.1007/s00464-021-08667-9 34312724 PMC8313000

[29] Neufeld M Bauerle W Eriksson E Azar F Evans H Johnson M Where did the patients go? Changes in acute appendicitis presentation and severity of illness during the coronavirus disease 2019 pandemic: a retrospective cohort study. Surgery. (2021) 169(4):808–15. 10.1016/j.surg.2020.10.035 33288212 PMC7717883

